# Healthy plant-based diet index as a determinant of bone mineral density in osteoporotic postmenopausal women: A case-control study

**DOI:** 10.3389/fnut.2022.1083685

**Published:** 2023-01-12

**Authors:** Marzieh Ghadiri, Elhameh Cheshmazar, Zainab Shateri, Shirin Gerami, Mehran Nouri, Bahram Pourghassem Gargari

**Affiliations:** ^1^Nutrition Research Center, Student Research Committee, Department of Biochemistry and Diet Therapy, Faculty of Nutrition and Food Sciences, Tabriz University of Medical Sciences, Tabriz, Iran; ^2^Department of Nutrition, School of Public Health, Iran University of Medical Sciences, Tehran, Iran; ^3^Student Research Committee, Ahvaz Jundishapur University of Medical Sciences, Ahvaz, Iran; ^4^Nutrition Research Center, School of Nutrition and Food Sciences, Shiraz University of Medical Sciences, Shiraz, Iran; ^5^Health Policy Research Center, Institute of Health, Shiraz University of Medical Sciences, Shiraz, Iran; ^6^Student Research Committee, Shiraz University of Medical Sciences, Shiraz, Iran; ^7^Nutrition Research Center, Department of Biochemistry and Diet Therapy, Faculty of Nutrition and Food Sciences, Tabriz University of Medical Sciences, Tabriz, Iran

**Keywords:** plant-based diet index, healthy plant-based diet index, unhealthy plant-based diet index, bone mass density, osteopenia

## Abstract

**Introduction:**

The association between plant-based diet indices and bone mineral density (BMD) of women with osteoporosis have not been studied in Iranian women. This study aimed to evaluate the association between plant-based diet indices and BMD in postmenopausal women with osteopenia/osteoporosis.

**Materials and methods:**

The present research was a case-control study conducted on 131 postmenopausal women with osteoporosis/osteopenia and 131 healthy women. The BMD of the femoral neck and lumbar vertebrae was measured by the Dual-energy X-ray absorptiometry (DXEA) method. Participants were asked to complete a validated semi-quantitative food frequency questionnaire (FFQ). We used three versions of plant-based diet indices, including plant-based diet index (PDI), healthy plant-based diet index (hPDI), and unhealthy plant-based diet index (uPDI). Two different multivariable logistic regression was used for the crude and adjusted model to assess the relationship between PDI, hPDI, and uPDI with odds of femoral and lumbar BMD.

**Results:**

There was a reverse association between last tertile of hPDI with femoral BMD abnormality in the both adjusted model [Model 1: odds ratio (OR): 0.33; 95% confidence interval (CI): 0.19–0.63 and Model 2: OR: 0.30; 95% CI: 0.15–0.58, respectively]. Furthermore, we found a reverse relationship between hPDI with lumbar BMD abnormality in the first adjusted model (OR: 0.36; 95% CI: 0.19–0.67). On the other hand, a negative association was observed in the second and last tertile of hPDI with lumbar BMD abnormality (OR: 0.47; 95% CI: 0.24–0.90 and OR: 0.34; 95% CI: 0.17–0.64, respectively). According to the results, the association of femoral BMD abnormality in the last tertile of uPDI compared to the first tertile in the both adjusted models (Model 1: OR: 2.85; 95% CI: 1.52–5.36 and Model 2: OR: 2.63; 95% CI: 1.37–5.06) were significant. Also, we observed a positive relationship between the last tertile of uPDI with lumbar BMD abnormality compared to the lowest tertile in the both adjusted models (Model 1; OR: 4.16; 95% CI: 2.20–7.85, Model 2; OR: 4.23; 95% CI: 2.19–8.19).

**Conclusion:**

Overall, the findings indicated that in postmenopausal women with osteoporosis, a healthy plant-based diet could prevent bone loss, and an unhealthy plant-based diet might have detrimental effects on BMD.

## Introduction

Osteoporosis (OP) is characterized by a decrease in bone mineral density (BMD) (1), which in turn reduces bone strength and makes it susceptible to fracture (2). OP usually develops gradually and does not show symptoms until a fracture occurs (3). Although OP can affect all bones, the bones of the pelvis, ribs, lumbar vertebrae, and wrists are most commonly affected (4). The worldwide prevalence of OP is reported to be 18.3% (5). Also, the prevalence of this disease in Iranian elderly is estimated at 41.5% (6).

It has been observed that the average and maximum amount of bone mass in women is lower than in men (7). One of the reasons can be hormonal changes that cause a 40–50% decrease in maximum bone mass in women (8). However, OP affects both genders of all ages. A reduction in BMD is more common in women with the onset of menopause, which occurs around age 50 (7).

Osteoporosis (OP) is a multifactorial disease (9). Lifestyle is one of the most critical factors affecting bone density. Among the lifestyle factors, nutrition plays an essential role in bone health (10). Intake of fruits, vegetables, calcium, potassium, magnesium, vitamins D and K can help bone health (10).

Plant-based diets have become popular due to the belief that healthier diets prevent chronic diseases (11). Some studies have investigated the relationship between plant-based diets and BMD (12, 13). Plant-based diets are rich in potassium, magnesium, vitamins C and K (10, 11), and other essential nutrients in bone matrix synthesis. Although the source of plant-based dietary proteins (such as legumes, grains, nuts, seeds, vegetables, etc.) provides low biological value, a recent systematic review found no significant difference in the consumption of plant and animal protein on bone health (14). On the other hand, a plant-based diet is generally lower in saturated fat and cholesterol, and increased dietary fiber and many phytochemicals promote bone health (15).

In addition, a meta-analysis study found that participants who followed a vegetarian diet had a 27% lower risk of OP (8). Contrary to the mentioned study, one study showed that the prevalence of OP was higher in Chinese postmenopausal women who frequently consumed vegetables (16). Also, in some studies, no association was observed between the consumption of a plant-based diet and the risk of developing OP (17, 18).

So, further studies are needed to demonstrate the association between the plant-based diet and OP. To the best of our knowledge, there is little information on the relationship between plant-based diet indices and BMD in Iranian postmenopausal women with OP/osteopenia. Therefore, this study investigated the relationship between these two variables in Iranian postmenopausal women with OP/osteopenia.

## Materials and methods

### Study population

**FIGURE 1:**
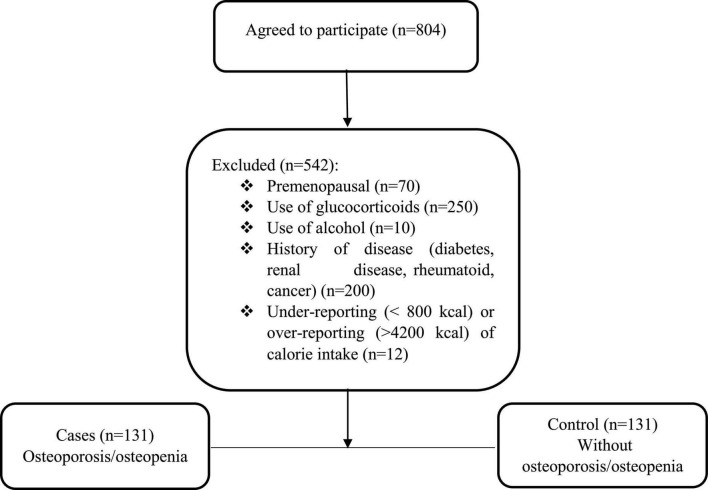
Flow chart of the study.

The present research was a case-control study conducted on 131 postmenopausal women with OP/osteopenia and 131 healthy postmenopausal women aged 45–65 years who were admitted to the Bone Densitometry Center in Isfahan, Iran (May 2021 to Dec 2021). The sample size was calculated based on the previous study considering OR = 2.30 (19). The exclusion criteria were premenopausal, use of glucocorticoids, alcohol, diabetes, renal disease, rheumatoid, cancer, and history of chemotherapy (Figure 1). Menopause was the absence of a menstrual cycle in the last 12 months.

The height was measured without shoes using a stadiometer and the stretch stature method with an accuracy of 0.5 cm. Also, body weight was measured without shoes and with the least clothes by a digital scale and recorded with an accuracy of 100 g. In addition, body mass index (BMI) was calculated as weight divided by height (square meters). A general information questionnaire collected information on socio-demographic, confounding, and contextual variables such as socioeconomic status and taking of drugs and supplements that affect BMD.

### Bone mineral density measurement

Bone mineral density (BMD) of the femoral neck and lumbar vertebrae in grams per square centimeter was measured by a technician using the Dual-energy X-ray absorptiometry (DXEA) method, and bone mass status was determined by a physician [device model: Horizon Wi (S/N 200451)]. Bone mass status was determined based on World Health Organization (WHO) criteria, according to which T-score values greater than −1 indicate normal bone mass, between −1 and −2.5 indicate osteopenia, and less than −2.5 indicate osteoporosis (20). Case samples were selected from individuals diagnosed with osteopenia and osteoporosis by a physician. But, control participants were selected from healthy postmenopausal volunteers with normal bone mass and other inclusion criteria who referred to the Bone Densitometry Center in Isfahan at the same time.

Information about the level of physical activity was collected using the International Physical Activity Questionnaire (IPAQ) (21). Physical activity of the participants based on MET (metabolic equivalent of task)-minutes and using the standard protocol were divided into three physical activity classes including low activity (below 600 MET-minutes/week), moderate (between 600 and 3,000 MET-minutes/week), and intense activity (above 3,000 MET-minutes/week).

### Dietary assessment and food grouping

Participants were asked to complete a validated semi-quantitative food frequency questionnaire (FFQ) (22) that included questions on their habitual daily consumption of food items during the past year. We used previous method to create three versions of plant-based diets, including plant-based diet index (PDI), healthy plant-based diet index (hPDI), and unhealthy plant-based diet index (uPDI) (23–25). All foods reported through the FFQ were classified into 18 food groups with three main classes: healthy plant foods (i.e., whole grains, fruits, vegetables, nuts, legumes, vegetable oils, and tea/coffee), unhealthy plant foods (i.e., fruit juices, sugar-sweetened beverages, refined grains, potatoes, and sweets/desserts), and animal foods (i.e., animal fat, dairy, egg, fish/seafood, meat, and miscellaneous animal-based foods). In PDI and hPDI, the highest consumption of plant foods and healthy plant foods got 10 scores, and the lowest consumption got 1 score. For unhealthy, a plant food score of 1 was considered for the highest consumption and 10 scores for the lowest consumption of unhealthy plant food. Scores for each of the PDI, hPDI, and uPDI were summed to obtain a score ranging from 18 to 180. A higher total score for each index indicated higher adherence to that dietary pattern.

### Ethics statement

The Ethics Committee of Tabriz University of Medical Sciences approved the protocols and procedures (Ethical Approval code: IR.TBZMED.REC.1400.114), and informed written consent was obtained from all participants after being informed about the purpose of this research.

### Statistical analysis

Participants were classified based on tertiles of PDI, hPDI, and uPDI. First, we used the residual method to adjust all the consumed food item (26). The Kolmogorov-Smirnov test was used to examine the normal distribution of the data. The baseline characteristics of the participants were described by means and standard deviations (SD) for the continuous variables and frequencies (percentages) for the categorical variables. Independent samples *T*-test and chi-square test were used to compare the continuous and categorical variables, respectively. Also, the ANOVA test was used for nutrient and food group analysis. Two different multivariable logistic regression were used for the crude and adjusted models to assess the relationship between PDI, hPDI, and uPDI with the odds of femoral and lumbar abnormality. We controlled the effects of BMI and age in the first model. Income, education, physical activity, and taking calcium supplements were added to the second model. Statistical analyses were carried out using SPSS (version 20.0, Inc., Chicago, IL, USA). *P*-value < 0.05 was considered statistically significant.

## Results

Mean age, BMI, femoral and lumbar BMD, taking vitamin D supplements, and demographic data of the case and control groups are shown in Table 1. According to the results, age (*P* = 0.03), femoral and lumbar BMD (*P* < 0.001 for both), physical activity level (*P* = 0.01), education level (*P* < 0.001), and vitamin D use (*P* = 0.01) was different between the case and control groups.

**TABLE 1:** Baseline characteristics of the study participants.

Variables	Control (*n* = 131)	Case (*n* = 131)	*P*-value
Age (year)	56.47 ± 5.91	57.95 ± 5.42	**0.03**
BMI (kg/m^2^)	29.13 ± 3.31	29.78 ± 3.99	0.15
BMD femoral (g/cm^2^)	0.78 ± 0.07	0.64 ± 0.09	**<0.001**
BMD lumbar (g/cm^2^)	1.00 ± 0.08	0.81 ± 0.09	**<0.001**
Income, average (%)	53 (40.5)	65 (49.6)	0.08
Physical activity, moderate (%)	22 (16.8)	9 (6.9)	**0.01**
Education level (%) Under diploma Diploma Higher diploma	65 (49.6) 52 (39.7) 14 (910.7)	98 (74.8) 25 (19.1) 8 (6.1)	**<0.001**
Calcium supplement (%) Yes No	32 (24.4) 99 (75.6)	32 (24.4) 99 (75.6)	0.55
Vitamin D supplement (%) Yes No	76 (58.0) 55 (42.0)	58 (44.3) 73 (55.7)	**0.01**

BMI, body mass index; BMD, bone mineral density.

Values are shown as mean for continuous and percentage for categorical variables.

Using independent samples *T*-test for continuous and chi-square test for categorical variables. Significant *p*-values are indicated in bold.

Nutrient intake between tertiles of PDI, hPDI, and uPDI are reported in Table 2. Intakes of energy (*P* < 0.001), carbohydrate (*P* < 0.001), fat (*P* < 0.001), fiber (*P* = 0.001), MUFA (*P* < 0.001), PUFA (*P* < 0.001), vitamin E (*P* < 0.001), folate (*P* < 0.001), sodium (*P* < 0.001), magnesium (*P* < 0.001), iron (*P* < 0.001), selenium (*P* < 0.001), copper (*P* < 0.001), phosphorus (*P* = 0.036), and zinc (*P* = 0.02) was higher in the last tertile of PDI compared to the first tertile.

**TABLE 2:** Nutrients intakes between tertiles of plant-based diet index (PDI), healthy plant-based diet index (hPDI), and unhealthy plant-based diet index (uPDI).

Variables	PDI				hPDI				uPDI			
	T1 (*n* = 88)	T2 (*n* = 85)	T3 (*n* = 88)	*P*	T1 (*n* = 90)	T2 (*n* = 87)	T3 (*n* = 84)	*P*	T1 (*n* = 88)	T2 (*n* = 83)	T3 (*n* = 90)	*P*
Energy (kcal/d)	1961.95 ± 247.88	2161.47 ± 337.90	2257.15 ± 409.28	**<0**.**001**	2219.61 ± 376.18	2104.17 ± 362.39	2050.18 ± 316.83	**0**.**006**	2324.99 ± 400.36	2100.44 ± 276.48	1959.64 ± 287.00	**<0**.**001**
Carbohydrate (g/day)	286.81 ± 36.84	319.59 ± 49.66	336.79 ± 56.70	**<0**.**001**	325.19 ± 58.96	313.30 ± 52.00	303.92 ± 43.44	**0**.**02**	399.07 ± 57.96	310.57 ± 42.46	294.10 ± 45.90	**<0**.**001**
Protein (g/day)	65.55 ± 11.93	68.17 ± 11.81	67.90 ± 14.95	0.34	68.29 ± 11.77	66.00 ± 13.53	67.26 ± 13.74	0.50	77.35 ± 12.64	67.98 ± 9.15	56.67 ± 6.79	**<0**.**001**
Fat (g/day)	68.41 ± 8.80	75.34 ± 14.35	78.69 ± 16.92	**<0**.**001**	78.45 ± 14.65	73.03 ± 15.07	70.65 ± 12.23	**0**.**001**	81.75 ± 15.96	72.85 ± 11.52	67.99 ± 11.72	**<0**.**001**
Fiber (g/day)	29.41 ± 6.38	30.98 ± 4.95	32.83 ± 6.32	**0**.**001**	28.48 ± 4.96	30.90 ± 5.25	34.09 ± 6.65	**<0**.**001**	36.35 ± 5.79	30.39 ± 4.13	26.61 ± 3.35	**<0**.**001**
SFA (g/day)	18.20 ± 3.92	18.80 ± 4.44	19.15 ± 6.09	0.43	20.02 ± 4.59	18.26 ± 5.19	17.78 ± 4.70	**0**.**006**	21.63 ± 5.20	18.80 ± 4.09	15.82 ± 3.44	**<0**.**001**
MUFA (g/day)	25.11 ± 3.07	27.03 ± 4.56	28.08 ± 5.86	**<0**.**001**	27.33 ± 4.40	26.64 ± 5.31	26.21 ± 4.61	0.29	29.55 ± 5.85	26.46 ± 3.69	24.29 ± 2.67	**<0**.**001**
PUFA (g/day)	17.52 ± 2.98	18.89 ± 3.71	20.47 ± 3.67	**<0**.**001**	19.33 ± 3.84	19.11 ± 3.52	26.21 ± 4.61	0.23	20.74 ± 4.16	18.58 ± 3.38	17.61 ± 2.59	**<0**.**001**
Vitamin A (RAE/day)	464.05 ± 265.35	481.53 ± 255.65	508.93 ± 293.57	0.54	432.59 ± 207.29	461.34 ± 178.98	566.18 ± 377.31	**0**.**003**	677.67 ± 337.66	452.03 ± 156.62	328.66 ± 140.59	**<0**.**001**
Vitamin E (mg/day)	20.87 ± 4.09	21.77 ± 4.33	23.63 ± 4.73	**<0**.**001**	21.75 ± 4.77	22.62 ± 4.00	21.94 ± 4.78	0.40	23.31 ± 5.09	21.50 ± 4.10	21.47 ± 4.13	**0**.**009**
Vitamin K (μg/day)	123.60 ± 74.79	123.50 ± 70.15	142.60 ± 80.40	0.15	113.53 ± 57.30	123.45 ± 54.06	154.70 ± 102.02	**0**.**001**	169.72 ± 89.67	126.14 ± 63.24	95.18 ± 48.59	**<0**.**001**
Vitamin B_6_ (mg/day)	1.64 ± 0.36	1.70 ± 0.31	1.73 ± 0.39	0.19	1.64 ± 0.30	1.67 ± 0.33	1.76 ± 0.42	0.05	1.98 ± 0.37	1.66 ± 0.23	1.43 ± 0.17	**<0**.**001**
Folate (μg/day)	427.99 ± 71.79	465.38 ± 69.25	498.01 ± 83.92	**<0**.**001**	474.71 ± 79.41	457.38 ± 79.04	458.95 ± 82.66	0.28	500.72 ± 88.87	456.18 ± 61.03	435.35 ± 74.33	**<0**.**001**
Vitamin B_12_ (μg/day)	2.88 ± 1.08	2.98 ± 1.43	2.79 ± 1.69	0.68	3.14 ± 1.37	2.95 ± 1.59	2.55 ± 1.24	**0**.**02**	3.61 ± 1.62	3.05 ± 1.15	2.04 ± 0.95	**<0**.**001**
Vitamin C (mg/day)	137.52 ± 66.41	137.98 ± 68.13	153.21 ± 75.41	0.24	118.37 ± 62.76	142.88 ± 57.01	169.80 ± 80.55	**<0**.**001**	196.23 ± 76.98	135.91 ± 47.24	97.99 ± 41.05	**<0**.**001**
Vitamin D (μg/day)	1.09 ± 0.80	0.78 ± 0.74	0.60 ± 0.59	**<0**.**001**	0.93 ± 0.79	0.78 ± 0.76	0.72 ± 0.63	**0**.**203**	1.08 ± 0.79	1.00 ± 0.80	0.41 ± 0.36	**<0**.**001**
Sodium (mg/day)	3499.82 ± 412.96	3664.86 ± 525.91	3888.30 ± 579.82	**<0**.**001**	3863.37 ± 564.62	3599.56 ± 509.74	3581.28 ± 477.37	**<0**.**001**	3815.11 ± 595.41	3598.98 ± 441.12	3638.57 ± 530.06	**0**.**01**
Calcium (mg/day)	511.09 ± 275.79	490.20 ± 251.98	466.16 ± 360.78	0.61	475.28 ± 253.04	458.98 ± 299.20	535.12 ± 342.90	0.21	705.79 ± 301.86	501.55 ± 243.30	268.05 ± 153.57	**<0**.**001**
Magnesium (mg/day)	390.43 ± 71.38	418.62 ± 61.09	439.97 ± 80.52	**<0**.**001**	403.94 ± 73.10	410.13 ± 70.96	436.41 ± 75.37	**0**.**009**	473.77 ± 67.50	418.10 ± 60.50	359.38 ± 41.41	**<0**.**001**
Phosphorus (mg/day)	2953.33 ± 742.80	3200.72 ± 759.01	3297 ± 924.99	**0**.**036**	3008.40 ± 721.25	3042.03 ± 751.09	3417.48 ± 931.17	**0**.**003**	3809 ± 798.74	3181.24 ± 645.23	2523.86 ± 398.38	**<0**.**001**
Iron (mg/day)	13.99 ± 1.89	15.31 ± 1.70	16.18 ± 2.229	**<0**.**001**	15.34 ± 2.21	14.94 ± 2.19	15.21 ± 2.12	0.46	16.36 ± 2.48	15.07 ± 1.69	14.10 ± 1.59	**<0**.**001**
Selenium (μ/day)	116.65 ± 16.60	123.02 ± 16.38	129.83 ± 18.30	**<0**.**001**	125.38 ± 17.69	121.42 ± 18.63	122.69 ± 17.39	0.32	129.65 ± 20.68	124.03 ± 16.63	116.20 ± 13.25	**<0**.**001**
Zinc (mg/day)	10.46 ± 2.05	11.22 ± 2.19	11.35 ± 2.58	**0**.**02**	11.27 ± 2.48	10.77 ± 2.17	10.99 ± 2.26	0.34	12.48 ± 2.14	11.26 ± 2.07	9.36 ± 1.50	**<0**.**001**
Copper (mg/day)	1.45 ± 0.24	**1**.**60** ± 0.25	1.68 ± 0.30	**<0**.**001**	1.57 ± 0.29	1.57 ± 0.30	1.59 ± 0.25	0.91	1.77 ± 0.27	1.58 ± 0.24	1.38 ± 0.18	**<0**.**001**

PDI, plant-based diet index; hPDI, healthy plant-based diet index; uPDI, unhealthy plant-based diet index; SFA, saturated fatty acid; PUFA, polyunsaturated fatty acid; MUFA, monounsaturated fatty acid; RAE, retinol activity equivalents.

Values are presented as mean ± SD.

Using one-way ANOVA. Significant *p*-values are indicated in bold.

In the last tertile of hPDI, the consumption of fiber (*P* < 0.001), vitamin C (*P* < 0.001), vitamin K (*P* = 0.001), vitamin A (*P* = 0.003), phosphorus (*P* = 0.003), and magnesium (*P* = 0.009) was higher compared to the first tertile, but intakes of energy (*P* = 0.006), carbohydrate (*P* = 0.02), fat (*P* = 0.001), sodium (*P* < 0.001), saturated fatty acid (22) (*P* < 0.006), and vitamin B_12_ (*P* = 0.02) was lower in the last tertile (Table 2). In the uPDI group, the consumption of all nutrients [vitamin E (*P* = 0.009), sodium (*P* = 0.01), and other nutrients (*P* < 0.001)] were lower in the last tertile than in the first tertile (Table 2).

The intake of food groups among tertiles of PDI, hPDI, and uPDI were presented in Table 3. According to Table 3, the consumption of whole (*P* = 0.001) and refined grains (*P* = 0.003), legumes (*P* = 0.03), vegetable oils (*P* < 0.001), tea/coffee (*P* < 0.001), fruit juice (*P* = 0.003), potato (*P* < 0.001), sugar-sweetened beverages (*P* < 0.001), sweets and desserts (*P* < 0.001) of participants in the last tertile of PDI was significantly higher, but intakes of dairy (*P* = 0.03), fish and seafood (*P* = 0.01) were lower in the last tertile compared to the first tertile.

**TABLE 3:** The intake of food groups between tertiles of plant-based diet index (PDI), healthy plant-based diet index (hPDI), and unhealthy plant-based diet index (uPDI).

Variables	PDI				hPDI				uPDI			
	T1 (*n* = 88)	T2 (*n* = 85)	T3 (*n* = 88)	*P*	T1 (*n* = 90)	T2 (*n* = 87)	T3 (*n* = 84)	*P*	T1 (*n* = 88)	T2 (*n* = 83)	T3 (*n* = 90)	*P*
Whole grains (g/day)	200.15 ± 45.20	211.35 ± 43.33	226.98 ± 49.45	**0**.**001**	194.84 ± 39.93	213.30 ± 46.73	232.04 ± 47.98	**<0**.**001**	226.53 ± 50.09	210.94 ± 49.34	201.49 ± 38.93	**0**.**002**
Fruits (g/day)	443.75 ± 203.74	443.41 ± 197.47	469.34 ± 210.85	0.62	369.47 ± 178.37	452.78 ± 165.30	541.65 ± 228.41	**<0**.**001**	604.35 ± 207.80	439.53 ± 144.01	317.01 ± 136.12	**<0**.**001**
Vegetables (g/day)	235.82 ± 118.26	232.32 ± 110.12	241.03 ± 120.84	0.88	198.83 ± 85.97	231.10 ± 98.20	282.76 ± 143.86	**<0**.**001**	322.35 ± 129.27	216.97 ± 81.23	171.15 ± 71.50	**<0**.**001**
Nuts (g/day)	8.11 ± 1.36	12.42 ± 1.37	9.92 ± 1.25	0.07	11.60 ± 1.85	9.43 ± 0.88	9.22 ± 0.96	0.37	13.18 ± 1.05	11.94 ± 1.55	5.50 ± 1.25	**<0**.**001**
Legumes (g/day)	23.77 ± 16.64	26.73 ± 12.03	29.48 ± 14.35	**0**.**03**	24.91 ± 13.10	24.35 ± 11.91	30.98 ± 17.65	**0**.**004**	32.85 ± 16.62	26.78 ± 13.09	20.58 ± 11.05	**<0**.**001**
Vegetable oils (g/day)	26.82 ± 5.10	29.24 ± 6.04	32.02 ± 5.56	**<0**.**001**	29.27 ± 6.17	30.38 ± 6.03	28.44 ± 5.52	0.10	30.56 ± 7.65	28.22 ± 5.30	29.27 ± 4.23	**0**.**03**
Tea and coffee (g/day)	584.82 ± 326.66	770.48 ± 374.91	948.74 ± 444.43	**<0**.**001**	757.74 ± 396.28	758.08 ± 417.55	791.47 ± 427.11	0.82	735.38 ± 360.80	729.64 ± 353.02	836.46 ± 496.05	0.15
Fruit juices (g/day)	1.36 ± 0.41	3.43 ± 0.85	7.26 ± 1.87	**0**.**003**	4.40 ± 0.86	4.49 ± 1.12	3.10 ± 1.69	0.68	3.37 ± 0.83	5.76 ± 1.87	3.05 ± 0.86	0.25
Refined grains	207.84 ± 82.71	244.01 ± 96.70	256.79 ± 108.47	**0**.**003**	290.56 ± 97.85	235.42 ± 75.78	178.14 ± 86.57	**<0**.**001**	199.65 ± 91.16	236.47 ± 76.85	271.30 ± 110.19	**<0**.**001**
Potatoes (g/day)	10.30 ± 1.05	15.59 ± 1.14	19.68 ± 1.32	**<0**.**001**	21.46 ± 1.25	15.29 ± 1.03	8.33 ± 1.01	**<0**.**001**	13.45 ± 1.48	14.77 ± 1.12	17.29 ± 1.08	0.08
Sugar-sweetened beverages (g/day)	8.91 ± 2.15	15.81 ± 2.47	31.85 ± 6.05	**<0**.**001**	30.07 ± 4.20	23.05 ± 5.21	2.64 ± 1.02	**<0**.**001**	13.12 ± 4.65	19.83 ± 3.51	23.76 ± 4.06	0.17
Sweets and desserts (g/day)	23.95 ± 15.00	41.29 ± 37.91	41.18 ± 21.73	**<0**.**001**	49.58 ± 38.68	33.76 ± 21.64	21.83 ± 13.86	**<0**.**001**	31.42 ± 31.24	32.20 ± 19.14	42.25 ± 29.41	**0**.**01**
Animal fat (g/day)	2.36 ± 0.42	2.91 ± 0.39	1.86 ± 0.36	0.17	3.90 ± 0.50	2.33 ± 0.34	0.76 ± 0.18	**<0**.**001**	2.51 ± 0.43	2.68 ± 0.41	1.95 ± 0.34	0.39
Dairy (g/day)	279.22 ± 141.56	246 ± 142.58	215.57 ± 194.68	**0**.**03**	250.79 ± 140.82	239.57 ± 172.14	250.58 ± 177.85	0.87	340.01 ± 173.88	266.04 ± 146.49	139.68 ± 90.44	**<0**.**001**
Egg (g/day)	14.21 ± 6.81	11.95 ± 6.56	12.85 ± 7.42	0.10	15.39 ± 6.67	11.91 ± 6.53	11.58 ± 7.16	**<0**.**001**	14.39 ± 7.77	13.43 ± 6.25	11.32 ± 6.52	**0**.**01**
Fish and seafood (g/day)	4.79 ± 0.64	3.70 ± 0.60	2.54 ± 0.26	**0**.**01**	3.17 ± 0.32	3.43 ± 0.38	4.47 ± 0.79	0.19	6.54 ± 0.77	2.97 ± 0.28	1.54 ± 0.16	**<0**.**001**
Meat (g/day)	37.03 ± 12.59	39.66 ± 11.80	36.22 ± 15.81	0.21	40.85 ± 13.76	38.55 ± 14.09	33.12 ± 11.62	**0**.**001**	42.71 ± 14.74	38.00 ± 10.55	32.32 ± 12.98	**<0**.**001**
Animal-based foods (g/day)	4.77 ± 0.58	5.56 ± 0.49	5.90 ± 0.56	0.33	8.40 ± 0.60	4.98 ± 0.47	2.62 ± 0.31	**<0**.**001**	5.74 ± 0.56	5.85 ± 0.53	4.69 ± 0.55	0.25

PDI, plant-based diet index; hPDI, healthy plant-based diet index; uPDI, unhealthy plant-based diet index.

Values are presented as mean ± SD.

Using one-way ANOVA. Significant *p*-values are indicated in bold.

Individuals in the last tertile of hPDI had higher intakes of whole grains (*P* < 0.001), fruits (*P* < 0.001), vegetables (*P* < 0.001), and legumes (*P* = 0.004) compared to the first tertile. In contrast, consumption of refined grains (*P* < 0.001), potato (*P* < 0.001), sugar-sweetened beverages (*P* < 0.001), sweets and desserts (*P* < 0.001), animal fat (*P* < 0.001), egg (*P* < 0.001), meats (*P* = 0.001), and animal-based foods (*P* < 0.001) was lower than the first tertile (Table 3).

The lower intakes of whole grains (*P* = 0.002), fruits (*P* < 0.001), vegetables (*P* < 0.001), nuts (*P* < 0.001), legumes (*P* < 0.001), vegetable oils (*P* = 0.03), dairy (*P* < 0.001), egg (*P* = 0.01), fish and seafood (*P* < 0.001), meat (*P* < 0.001), and higher intakes of refined grains (*P* < 0.001), sweets, and desserts (*P* = 0.01) was seen in the participants of the last tertile compared to the first tertile of uPDI group (Table 3).

According to Table 4, there was no relationship between PDI and femoral and lumbar BMD abnormalities in the crude and both adjusted models. As shown in Table 4, there was a reverse association between the second and last tertile of hPDI with femoral BMD abnormality in the crude model [odds ratio (OR): 0.43; 95% confidence interval (CI): 0.23–0.80 and OR: 0.35; 95% CI: 0.19–0.66, respectively] and both adjusted model (Model 1: OR: 0.38; 95% CI: 0.20–0.72 and OR: 0.33; 95% CI: 0.19–0.63, respectively and Model 2: OR: 0.31; 95% CI: 0.15–0.61, and OR: 0.30; 95% CI: 0.15–0.58, respectively).

**TABLE 4:** Crude and multivariable-adjusted odds ratios and 95% CIs across tertile of plant-based diet index (PDI), healthy plant-based diet index (hPDI), and unhealthy plant-based diet index (uPDI).

Variables	Femoral BMD abnormality			Lumbar BMD abnormality		
	Crude	Adjusted model 1	Adjusted model 2	Crude	Adjusted model 1	Adjusted model 2
**PDI**						
T1	Ref.	Ref.	Ref.	Ref.	Ref.	Ref.
T2	0.62 (0.34–1.14)	0.68 (0.36–1.26)	0.71 (0.37–1.36)	0.81 (0.44–1.47)	0.86 (0.47–1.58)	0.90 (0.48–1.69)
T3	0.75 (0.41–1.36)	0.80 (0.43–1.48)	0.74 (0.39–1.40)	1.11 (0.61, 2.02)	1.20 (0.65–2.20)	1.10 (0.57–2.06)
P_trend_	0.35	0.48	0.37	0.71	0.55	0.72
**hPDI**						
T1	Ref.	Ref.	Ref.	Ref.	Ref.	Ref.
T2	0.43 (0.23–0.80)	0.38 (0.20–0.72)	0.31 (0.15–0.61)	0.58 (0.31–1.06)	0.55 (0.30–1.01)	0.47 (0.24–0.90)
T3	0.35 (0.19–0.66)	0.33 (0.19–0.63)	0.30 (0.15–0.58)	0.36 (0.20, 0.68)	0.36 (0.19–0.67)	0.34 (0.17–0.64)
P_trend_	**0.001**	**0.001**	**< 0.001**	**0.001**	**0.001**	**0.001**
**uPDI**						
T1	Ref.	Ref.	Ref.	Ref.	Ref.	Ref.
T2	1.09 (0.59–1.99)	1.03 (0.55–1.90)	1.08 (0.57–2.04)	1.40 (0.76–2.58)	1.36 (0.74–2.52)	1.37 (0.73–2.59)
T3	2.64 (1.43–4.88)	2.85 (1.52–5.36)	2.63 (1.37–5.06)	3.97 (2.12–7.42)	4.16 (2.20–7.85)	4.23 (2.19–8.19)
P_trend_	**0.002**	**0.001**	**0.004**	**< 0.001**	**< 0.001**	**< 0.001**

BMD, bone mass density; PDI, plant-based diet index; hPDI, healthy plant-based diet index; uPDI, unhealthy plant based diet index.

Model 1: adjusted for BMI and age.

Model 2: additionally, adjusted for income, education, physical activity, and calcium supplement.

These values are shown as odds ratio (95% CIs). Obtained from logistic regression. Significant *p*-values are indicated in bold.

Furthermore, we found a reverse relationship between hPDI with lumbar BMD abnormality in the crude model (OR: 0.36; 95% CI: 0.20–0.68) and the first adjusted model (OR: 0.36; 95% CI: 0.19–0.67). On the other hand, a negative association was observed in the second and last tertile of hPDI with lumbar BMD abnormality (OR: 0.47; 95% CI: 0.24–0.90 and OR: 0.34; 95% CI: 0.17–0.64, respectively).

According to the results, the association of femoral BMD abnormality in the last tertile of uPDI compared to the first tertile in the crude (OR: 2.64; 95% CI: 1.43–4.88) and both adjusted models (Model 1: OR: 2.85; 95% CI: 1.52–5.36 and Model 2: OR: 2.63; 95% CI: 1.37–5.06) were significant. Also, we observed a positive relationship between the last tertile of uPDI with lumbar BMD abnormality compared to the lowest tertile in the crude (OR: 3.97; 95% CI: 2.12–7.42) and both adjusted models (Model 1; OR: 4.16; 95% CI: 2.20–7.85, Model 2; OR: 4.23; 95% CI: 2.19–8.19).

## Discussion

In the present case-control study, it was shown that higher scores of PDI indicating a higher intake of whole grains, legumes, vegetable oil, tea and coffee, fruit juices, refined grains, potatoes, sugar-sweetened beverages, sweets, and desserts were not significantly related to BMD abnormality of the femoral neck and lumbar vertebrae. Previous studies have shown conflicting findings regarding the relationship between vegetarian diet and bone density (27). In a study conducted by Shahinfar et al., it was shown that there is no significant relationship between PDI and osteocalcin as a biomarker of bone formation among older adults (28). It has also been shown that vegetarians are not subjected to a higher risk of developing OP than non-vegetarians (29). In contrast, a review study demonstrated that a plant-based diet reduces the density of the femoral neck, lumbar spine, and whole body compared to an omnivorous diet (11).

Bone is a living tissue sensitive to body conditions. Subtle changes in acid-base balance and nutrient intake can alter bone metabolism and long-term bone density (30, 31). Decreased BMD is an important risk factor for fractures throughout life (32). A plant-based diet has positive effects on bones, including the consumption of fruits and vegetables, which help to maintain calcium in the body due to the presence of potassium (33). This diet has more antioxidants, phytochemicals, and vitamins and imposes less acid load on the body (34). Also, the negative effects of this diet include lower amounts of protein, higher phytic acid and as a result less absorption of zinc from the diet (35), and lower amounts of calcium and vitamin D, all of which are considered important for improving bone health (34, 36). The interaction between the positive and negative effects of a plant-based diet neutralizes each other, which may be why a plant-based diet does not significantly affect BMD. However, plant-based diets affect multiple inflammatory and systemic responses, such as microbiota metabolites (37, 38). There are findings in animal models that microbiota act as crucial mediators in bone remodeling (12). It is also shown that diet can alter the diversity of an individual’s microbiota in human models, suggesting a mechanism by which plant-based diets can affect bone health (12).

As shown in the current study, the higher hPDI scores are correlated with greater protective effects against the decrease in the femoral neck and lumbar vertebrae BMD. A study implemented by Shahinfar et al. it was indicated that there was no significant relationship between hPDI and osteocalcin (28). The present case-control study indicated that higher hPDI scores are associated with a higher intake of fruits and vegetables, which was statistically significant. It has been demonstrated that more fruits and vegetables are related to more BMD and OP risk reduction in middle-aged and older adults (39). This effect of fruits and vegetables may be because they are good potassium, calcium, and magnesium sources, which can neutralize the effects of calcium excretion in urine caused by dietary acid (40).

It was observed that uPDI significantly increases the chance of abnormality in BMD in both the femoral neck and lumbar vertebrae regions. As shown in the present study, uPDI is associated with a decrease in the intake of whole grains, fruits, vegetables, nuts, legumes, and dairy products, all of which contribute to increased bone density (15, 41, 42). The results of a study investigating the relationship between uPDI and osteocalcin showed that there is an inverse relationship between uPDI and osteocalcin (28). Osteocalcin plays an important role in regulating bone mineralization, osteoblast, and osteoclast activity (43). Therefore, an unhealthy plant-based diet can reduce the amounts of osteocalcin, which may reduce bone formation and BMD.

If we want to point out the limitations of the present study, we can mention examining dietary intake through the FFQ questionnaire, which depends on long-term memory. Also, this questionnaire can overestimate the intake of nutrients. However, FFQ is the most common dietary assessment method in epidemiological studies and is an easy and effective tool for collecting dietar y information.

Overall, the findings indicated a healthy plant-based diet could exert a protective effect in preventing bone loss. In contrast, an unhealthy plant-based diet can negatively affect BMD in postmenopausal OP women. Consuming proteins with high biological value, vegetables, fruits, legumes, whole grains, soy products, and nuts can help bone health. Therefore, properly planning a vegetarian diet is not only harmful to bone health but can also hold protective effects in preventing bone loss. Further studies are necessary to investigate the generalizability of these findings to other populations with different demographic or biological characteristics.

## Data availability statement

The raw data supporting the conclusions of this article will be made available by the authors, without undue reservation.

## Ethics statement

The studies involving human participants were reviewed and approved by the Research Ethics Committee of Tabriz University of Medical Sciences, Tabriz, Iran (IR.TBZMED.REC.1400.114). The patients/participants provided their written informed consent to participate in this study.

## Author contributions

EC, MG, ZS, and SG contributed to the data collection and writing the first draft. MN and SG contributed to the statistical analysis and interpretation of data. BG contributed in funding and project management. All authors read and approved the final manuscript.

## References

[1] Acosta A Dayyeh B Port J Camilleri M. Recent advances in clinical practice challenges and opportunities in the management of obesity. *Gut.* (2014) 63:687–95. 10.1136/gutjnl-2013-306235 24402654 PMC4170188

[2] Hernlund E Svedbom A Ivergård M Compston J Cooper C Stenmark J Osteoporosis in the European Union: medical management, epidemiology and economic burden. *Arch Osteoporos.* (2013) 8:136. 10.1007/s11657-013-0136-1 24113837 PMC3880487

[3] He J Xu S Zhang B Xiao C Chen Z Si F Gut microbiota and metabolite alterations associated with reduced bone mineral density or bone metabolic indexes in postmenopausal osteoporosis. *Aging.* (2020) 12:8583. 10.18632/aging.103168 32392181 PMC7244073

[4] Tarantino U Capone A Planta M D’Arienzo M Letizia Mauro G Impagliazzo A The incidence of hip, forearm, humeral, ankle, and vertebral fragility fractures in Italy: results from a 3-year multicenter study. *Arthritis Res Ther.* (2010) 12:R226. 10.1186/ar3213 21190571 PMC3046539

[5] Salari N Ghasemi H Mohammadi L Rabieenia E Shohaimi S Mohammadi M. The global prevalence of osteoporosis in the world: a comprehensive systematic review and meta-analysis. *J Orthop Surg Res.* (2021) 16:1–20. 10.1186/s13018-021-02772-0 34657598 PMC8522202

[6] Fahimfar N Noorali S Yousefi S Gharibzadeh S Shafiee G Panahi N Prevalence of osteoporosis among the elderly population of Iran. *Arch Osteoporos.* (2021) 16:1–10. 10.1007/s11657-020-00872-8 33475880

[7] Lim Y Lee S Tserendejid Z Jeong S Go G Park H. Prevalence of osteoporosis according to nutrient and food group intake levels in Korean postmenopausal women: using the 2010 Korea National Health and Nutrition Examination Survey Data. *Nutr Res Pract.* (2015) 9:539–46. 10.4162/nrp.2015.9.5.539 26425285 PMC4575968

[8] Zeng L Yang W Liang G Luo M Cao Y Chen H Can increasing the prevalence of vegetable-based diets lower the risk of osteoporosis in postmenopausal subjects? A systematic review with meta-analysis of the literature. *Complement Ther Med.* (2019) 42:302–11. 10.1016/j.ctim.2018.11.026 30670259

[9] Rosen C. *The Epidemiology and Pathogenesis of Osteoporosis.* South Dartmouth, MA: Endotext (2020).

[10] Hejazi J Davoodi A Khosravi M Sedaghat M Abedi V Hosseinverdi S Nutrition and osteoporosis prevention and treatment. *Biomed Res Ther.* (2020) 7:3709–20. 10.15419/bmrat.v7i4.598

[11] Ma X Tan H Hu M He S Zou L Pan H. The impact of plant-based diets on female bone mineral density: evidence based on seventeen studies. *Medicine.* (2021) 100:e27480. 10.1097/MD.0000000000027480 34797275 PMC8601298

[12] Hsu E. Plant-based diets and bone health: sorting through the evidence. *Curr Opin Endocrinol Diabetes Obes.* (2020) 27:248–52. 10.1097/MED.0000000000000552 32618637

[13] Smith A. Veganism and osteoporosis: a review of the current literature. *Int J Nurs Pract.* (2006) 12:302–6. 10.1111/j.1440-172X.2006.00580.x 16942519

[14] Shams-White M Chung M Fu Z Insogna K Karlsen M LeBoff M Animal versus plant protein and adult bone health: a systematic review and meta-analysis from the National Osteoporosis Foundation. *PLoS One.* (2018) 13:e0192459. 10.1371/journal.pone.0192459 29474360 PMC5825010

[15] Chuang T Lin C Wang Y. Effects of vegetarian diet on bone mineral density. *Tzu Chi Med J.* (2021) 33:128. 10.4103/tcmj.tcmj_84_20 33912409 PMC8059457

[16] Liu N Zeng F Zhang K Tang Z. A community-based cross-sectional study for relationship of frequency of vegetables intake and osteoporosis in a Chinese postmenopausal women sample. *BMC Womens Health.* (2016) 16:28. 10.1186/s12905-016-0307-5 27259804 PMC4891848

[17] Shin S Joung H. A dairy and fruit dietary pattern is associated with a reduced likelihood of osteoporosis in Korean postmenopausal women. *Br J Nutr.* (2013) 110:1926–33. 10.1017/S0007114513001219 23578480

[18] Shin A Lim S Sung J Myung S Kim J. Dietary habit and bone mineral density in Korean postmenopausal women. *Osteoporos Int.* (2010) 21:947–55. 10.1007/s00198-009-1039-2 19727908

[19] Shivappa N Hébert J Karamati M Shariati-Bafghi S Rashidkhani B. Increased inflammatory potential of diet is associated with bone mineral density among postmenopausal women in Iran. *Eur J Nutr.* (2016) 55:561–8. 10.1007/s00394-015-0875-4 25778389

[20] Cashman K. Diet, nutrition, and bone health. *J Nutr.* (2007) 137:2507S–12S. 10.1093/jn/137.11.2507S 17951494

[21] Barrientos-Gutierrez T Moore K Auchincloss A Mujahid M August C Sanchez B Neighborhood physical environment and changes in body mass index: results from the multi-ethnic study of atherosclerosis. *Am J Epidemiol.* (2017) 186:1237–45. 10.1093/aje/kwx186 29206987 PMC5860514

[22] Mirmiran P Esfahani F Mehrabi Y Hedayati M Azizi F. Reliability and relative validity of an FFQ for nutrients in the Tehran lipid and glucose study. *Public Health Nutr.* (2010) 13:654–62. 10.1017/S1368980009991698 19807937

[23] Borazjani M Nouri M Venkatakrishnane K Najafi M Faghih S. Association of plant-based diets with lipid profile and anthropometric indices: a cross-sectional study. *Nutr Food Sci.* (2022) 52:830–42. 10.1108/NFS-06-2021-0181 19194102

[24] Zamani B Daneshzad E Siassi F Guilani B Bellissimo N Azadbakht L. Association of plant-based dietary patterns with psychological profile and obesity in Iranian women. *Clin Nutr.* (2020) 39:1799–808. 10.1016/j.clnu.2019.07.019 31399262

[25] Nouri M Abdollahi N Leilami K Shirani M. The relationship between plant-based diet index and semen parameters of men with infertility: a cross-sectional study. *Int J Fertil Steril.* (2022) 16:310–9. 36273319 10.22074/IJFS.2021.538675.1184 PMC9627011

[26] Forman M. Nutritional epidemiology. 2nd ed. In: Willett W editor. *The American Journal of Clinical Nutrition.* (Vol. 69), New York, NY: Oxford University Press (1999). 1020 p. 10.1093/ajcn/69.5.1020

[27] Ho-Pham L Nguyen N Nguyen T. Effect of vegetarian diets on bone mineral density: a Bayesian meta-analysis. *Am J Clin Nutr.* (2009) 90:943–50. 10.3945/ajcn.2009.27521 19571226

[28] Shahinfar H Amini M Payandeh N Naghshi S Sheikhhossein F Djafarian K The link between plant-based diet indices with biochemical markers of bone turn over, inflammation, and insulin in Iranian older adults. *Food Sci Nutr.* (2021) 9:3000–14. 10.1002/fsn3.2258 34136166 PMC8194905

[29] Giudici K Weaver C. Plant-based diets and risk of osteoporosis. In: Craig W editor. *Vegetarian Nutrition and Wellness.* Boca Raton, FL: CRC Press (2018). p. 93–112. 10.1201/b22003-6

[30] Ahn H Kim J Lee K Kim H Jeong D. Extracellular acidosis accelerates bone resorption by enhancing osteoclast survival, adhesion, and migration. *Biochem Biophys Res Commun.* (2012) 418:144–8. 10.1016/j.bbrc.2011.12.149 22244876

[31] Arnett T Dempster D. Effect of pH on bone resorption by rat osteoclasts in vitro. *Endocrinology.* (1986) 119:119–24. 10.1210/endo-119-1-119 3720660

[32] Wu F Wills K Laslett L Oldenburg B Jones G Winzenberg T. Associations of dietary patterns with bone mass, muscle strength and balance in a cohort of Australian middle-aged women. *Br J Nutr.* (2017) 118:598–606. 10.1017/S0007114517002483 28990541

[33] Lemann J Jr Gray R Pleuss J. Potassium bicarbonate, but not sodium bicarbonate, reduces urinary calcium excretion and improves calcium balance in healthy men. *Kidney Int.* (1989) 35:688–95. 10.1038/ki.1989.40 2540373

[34] Burckhardt P. The role of low acid load in vegetarian diet on bone health: a narrative review. *Swiss Med Wkly.* (2016) 146:w14277. 10.4414/smw.2016.14277 26900949

[35] Harland B Oberleas D. Phytate in foods. *Energy Nutr Women.* (1987) 52:235–59. 10.1159/000415199 3327233

[36] McEvoy C Temple N Woodside J. Vegetarian diets, low-meat diets and health: a review. *Public Health Nutr.* (2012) 15:2287–94. 10.1017/S1368980012000936 22717188 PMC10271837

[37] Kim M Hwang S Park E Bae J. Strict vegetarian diet improves the risk factors associated with metabolic diseases by modulating gut microbiota and reducing intestinal inflammation. *Environ Microbiol Rep.* (2013) 5:765–75. 10.1111/1758-2229.12079 24115628

[38] Koeth R Lam-Galvez B Kirsop J Wang Z Levison B Gu X l-Carnitine in omnivorous diets induces an atherogenic gut microbial pathway in humans. *J Clin Invest.* (2019) 129:373–87. 10.1172/JCI94601 30530985 PMC6307959

[39] Qiu R Cao W Tian H He J Chen G Chen Y. Greater intake of fruit and vegetables is associated with greater bone mineral density and lower osteoporosis risk in middle-aged and elderly adults. *PLoS One.* (2017) 12:e0168906. 10.1371/journal.pone.0168906 28045945 PMC5207626

[40] Lambert H Frassetto L Moore J Torgerson D Gannon R Burckhardt P The effect of supplementation with alkaline potassium salts on bone metabolism: a meta-analysis. *Osteoporos Int.* (2015) 26:1311–8. 10.1007/s00198-014-3006-9 25572045

[41] Shin S Sung J Joung H. A fruit, milk and whole grain dietary pattern is positively associated with bone mineral density in Korean healthy adults. *Eur J Clin Nutr.* (2015) 69:442–8. 10.1038/ejcn.2014.231 25351648

[42] Shahriarpour Z Nasrabadi B Shariati-Bafghi S Karamati M Rashidkhani B. Adherence to the dietary approaches to stop hypertension (DASH) dietary pattern and osteoporosis risk in postmenopausal Iranian women. *Osteoporos Int.* (2020) 31:2179–88. 10.1007/s00198-020-05450-9 32556519

[43] Neve A Corrado A Cantatore F. Osteocalcin: skeletal and extra-skeletal effects. *J Cell Physiol.* (2013) 228:1149–53. 10.1002/jcp.24278 23139068

